# Antiulcerogenic Activity of the Hydroalcoholic Extract of Leaves of *Croton campestris* A. St.-Hill in Rodents

**DOI:** 10.1155/2013/579346

**Published:** 2013-06-20

**Authors:** Francisco E. B. Júnior, Dayanne R. de Oliveira, Elizângela B. Bento, Laura H. I. Leite, Daniele O. Souza, Ana Luiza A. Siebra, Renata S. Sampaio, Anita O. P. B. Martins, Andreza G. B. Ramos, Saulo R. Tintino, Luiz J. Lacerda-Neto, Patricia R. L. Figueiredo, Larissa R. Oliveira, Cristina K. S. Rodrigues, Valterlúcio S. Sales, Francisco R. S. D. N. Figueiredo, Emmily P. Nascimento, Alefe B. Monteiro, Érika N. Amaro, José G. M. Costa, Henrique Douglas Melo Coutinho, Irwin R. A. de Menezes, Marta R. Kerntopf

**Affiliations:** Department of Biological Chemistry, Regional University of Cariri, Crato, CE, Brazil

## Abstract

*Croton campestris* A. St.-Hill., popularly known as “velame do campo,” is a species native to the savannah area of Northeast Brazil, which is used by traditional communities in folk medicine for variety of health problems, especially detoxification, inflammation, and gastritis. The hydroalcoholic extract of *C. campestris* leaves (HELCC) was assessed for its antiulcerogenic effect in gastric lesion models and effect on intestinal motility in mice, and possible mechanisms of action were examined. HELCC showed significant gastroprotective action in all models of gastric ulcer evaluated; the results suggest that this action probably involves the nitric oxide pathway. HELCC did not show alteration of intestinal motility in mice. It was also found that *C. campestris* represents a promising natural source with important biological potential, justifying some of its uses in folk medicine.

## 1. Introduction

 Peptic ulcer is a term used to describe a group of ulcerative disorders that occur in areas of the upper gastrointestinal tract, which are exposed to acidic secretions and pepsin. It represents a chronic health problem. Approximately 10% of the population have or will develop peptic ulcer. Its incidence is slightly higher in men than women (1.3 : 1), and although it occurs at any age, duodenal ulcer occurs most often in the range of 30–55 years, whereas gastric ulcer occurs in the range of 50–70 years [1].

In Brazil, despite the absence of epidemiological records of ulcer cases, it is known that there are numerous cases involving this disease, which means a significant public health problem prompting the search for new substances with antiulcerogenic activity. Although there is a large arsenal of drugs with antiulcerogenic activity on the market, none produces 100% remission of ulcers, with reduced side effects and without compromising the patient's wellbeing, which usually results in chronic use of these drugs. Published studies have reported the widespread identification of new drugs derived from natural antiulcerogenic sources [2].

Studies have shown that lipid peroxidation and oxidative reactions are implicated in the pathogenesis of lesions induced by ethanol by attacking biological molecules and prostaglandins. Thus, research in animal models has attracted the interest of many investigators as a promising source for the discovery of new drugs [3–5]. 

 The genus *Croton*, whose name means “tick,” is the second largest in the family Euphorbiaceae and belongs to the subfamily *Crotonoidae Crotoneae* and tribe [6]. It is subdivided into 40 sections and has more than 1,300 species of mainly pantropical distribution, of which 350 species in 29 sections occur in Brazil [7]. Of these, a total of 52 species in 18 sections are referred to Northeast Brazil [8].

 Several species of *Croton* have long played an important role in traditional uses of medicinal plants in Africa, Asia, and South America, including the treatment of cancer, constipation, diarrhea, and other digestive problems, diabetes, external wounds, fever, hypercholesterolemia, hypertension, inflammation, intestinal worms, malaria, pain, ulcers, and obesity [9].

Studies involving several species of the genus *Croton* have shown different pharmacological activities such as anti-inflammatory and antioxidant activities of *C. celtidifolius* [10], antileishmanial effect of *C. cajucara* [11], antinociceptive effect of *C. cajucara* [12], antiulcerogenic and cytotoxic activities of *C. cajucara* [13], hypoglycemic and hypolipidemic properties of *C. cajucara* [14], antimicrobial and the antimalarial effects *C. kongensis* [15], antinociceptive and anti-inflammatory properties of *C. malambo* [16], antimicrobial effect of *C. sonderianus* [17], purgative effect of *C. campestris* [18], and hypotensive and narcotic activities of *C. eluteria* [19].

A study of *C. campestris *in the traditional community of the bioregion of Chapada do Araripe revealed the popular use of the leaves and roots of this plant for gastric, hematological, and inflammatory disturbances, wounds, and respiratory problems. For their medicinal uses, the local population utilizes infusion in alcoholic beverages, teas, and macerates, which justifies the use of the hydroalcoholic extract for the verification of the antiulcer activity.

 The hydroalcoholic extract of leaves of *C. campestris* A. St.-Hill (HELCC) was assessed for its antiulcerogenic effect in models of gastric lesions using absolute ethanol, acidified ethanol, and indomethacin, and mechanisms of action were examined. The effect of HELCC on gastric motility was also ruled out.

## 2. Materials and Methods

### 2.1. Plant Material

The leaves of the plant species *Croton campestris* A. St.-Hill were collected in the municipality of Crato, Ceará, Brazil, using a GPS device (7° 22′ 2.8′′ S; 39° 28′ 42.4′′  W; altitude: 892 meters). The procedures for specimen preparation followed the recommendations described by Ming 1996 *apud *Di Stasi, 1996. The material was deposited in the Herbarium of the Federal University of Rio Grande do Norte (UFRN) and registered under no. 7095. The research was reviewed by the Ethics Committee for Animal Research, CEPA, Faculty of Medicine of Juazeiro do Norte (FMJ), and was approved (case no. 2009_0432_FR 271610).

### 2.2. Preparation of Hydroalcoholic Extract of the Leaves of *Croton Campestris* A. St.-Hill (HELCC)

The plant material (fresh leaves: 852 g *C. campestris*) was washed under running water, crushed and macerated, and then submitted to cold extraction with 99.9% ethanol and water in a 1 : 1 proportion (8.7 L of solvent). The solvent was removed using a rotary evaporator under reduced pressure and temperature of 40–50°C, and a yield of 70.89 g crude extract was obtained using a lyophilizer.

### 2.3. Phytochemical Screening

The material was submitted to phytochemical screening for the presence of tannins, flavonoids, saponins, steroids, triterpenes, coumarins, quinones, organic acids, and alkaloids. Accordingly, the hydroalcoholic extract (0.3 g) was diluted in distilled water (9 mL) and 70% ethanol (21 mL) and after dilution, distributed into six flasks, in which the tests for different compounds were performed. Phenols and tannins: 3 drops of ferric chloride were added to the flask, and development of a blue color denoted the presence of pyrogallic tannins, which could be further confirmed with the addition of gelatin, resulting in a precipitate with the sample. Anthocyanins, anthocyanidins, and flavonoids: the sample was acidified (pH 3) by the addition of 18 drops of HCl, and no change in color of the flask demonstrated the presence of flavones, flavonols, xanthones, and flavanonols.

The test for leucoanthocyanidins, catechins, and flavonones was performed by alkalinization to pH 8.5 with 1% sodium hydroxide in the first flask, acidification (pH 3) by means of 18 drops of HCl and heating for 2 min in the second flask, and alkalinization (pH 11) with 18 drops of 1% NaOH and also heating for 2 min in the third flask. Thus, flask 2 did not show a change in color, demonstrating the presence of flavonones, and still the orange red color of flask 3 also revealed the presence of flavonones. The test for chalcones and aurones, by alkalinization (pH 11) with 18 drops of 1% NaOH, gave a purple red color in the flask, indicating the presence of these compounds. 

The test for alkaloids was done by diluting the extract (0.3 g) in 30 mL of 5% acetic acid and 15 mL of ammonia for alkalinization. The preparation was heated until boiling, and 10 mL of 10% ammonium hydroxide was then added, along with 15 mL of chloroform. After homogenization and allowing to stand in a separatory funnel, the chloroform phase was collected in a beaker, and the solvent was evaporated. The residue was resuspended in 1% HCl and homogenized, and solution was placed on a slide along with a drop of Dragendorff reagent, where the formation of a precipitate was indicative of the presence of alkaloids.

The test for steroids and triterpenoids was carried out by dilution of the extract (0.3 g) in 6 mL of chloroform and filtration using cotton covered with sodium sulfate, collecting the filtrate in a test tube. After the addition of 0.1 mL of anhydrous acetic acid and three drops of sulfuric acid, the development of a green color indicated the presence of steroids [20].

### 2.4. Assays

#### 2.4.1. Assessment of Oral Acute Toxicity

Swiss mice were used throughout to evaluate HELCC for toxicity and effect on gastric lesions. The animals were fasted for 3 h prior to the experiment and were given a single dose of extract dissolved in 2% w/v Tween 80 at 17.5, 55, 175, 550, 2000, and 5000 mg/kg and observed for mortality for up to 48 h (short-term toxicity). Based on the short-term toxicity, the dose of the next animal was determined as per OECD guideline 425. All animals were also observed for long-term toxicity (14 days). The LD50 of the test extracts was calculated using “AOT 425” software provided by the Environmental Protection Agency, USA [21].

### 2.5. Gastric Lesions Induced by Ethanol [22]

The mice were divided into groups (*n* = 6), fasted for a period of 15 h and treated with HELCC (50, 75, 125, 250, 500, and 750 mg/kg, p.o.), omeprazole (30 mg/kg, p.o.), or vehicle (saline, 0.1 mL/10 g, p.o.) 1 h before administration of absolute ethanol (0.2 mL/animal, p.o.). After 30 min, the animals were sacrificed by cervical dislocation. Their stomach was removed, opened along the greater curvature, and rinsed with saline and digitized; the ulcerated area was expressed as a percentage relative to the total area of the gastric body using ImageJ software.

### 2.6. Gastric Lesions Induced by Acidified Ethanol [23]

The mice were treated with HELCC (50, 75, 125, 250, 500, and 750 mg/kg, p.o.), omeprazole (30 mg/kg, p.o.), or vehicle (saline, 0.1 mL/10 g, p.o. for the control lesion group). One hour after treatment, the animals received 0.2 mL of 0.3 M hydrochloric acid (HCl) in 60% ethanol and were sacrificed 1 h later. The percentage of stomach ulceration was determined as described above.

### 2.7. Gastric Lesions Induced by Indomethacin [24]

The mice were pretreated with HELCC (250, 500, and 750 mg/kg, p.o.), omeprazole (30 mg/kg, p.o.), or vehicle (saline, 0.1 mL/10 g, p.o. for the control lesion group). Six hours after administration of the ulcerogenic agent (indomethacin, 10 mg/kg, s.c.), the animals were sacrificed. The percentage of stomach ulceration was determined as described above.

### 2.8. Evaluation of the Mechanism of Action of Gastroprotective HELCC

To determine the mechanism of action, experiments were performed to separately examine the involvement of *α*-2 receptors, prostaglandins [23], nitric oxide [25], and ATP-dependent K^+^ channel activation [26] in the gastroprotective effect of HELCC (75 mg/kg, p.o.). These experiments used the appropriate antagonists including yohimbine (2 mg/kg, i.p.), indomethacin (10 mg/kg, p.o.), L-NAME (10 mg/kg, i.p.), and glibenclamidee (5 mg/kg, p.o.) or agonists including L-arginine (600 mg/kg, p.o.) as a positive control for L-NAME and misoprostol (0.016 mg/kg, p.o.) as a control for indomethacin before the oral administration of 0.2 mL of 96% ethanol.

 In each case, the animals were pretreated with the specific antagonist or agonist for 30 min before the administration of HELCC. Finally, 0.2 mL of 96% ethanol was orally administered one hour after HELCC. The animals were then sacrificed, and their stomach was removed, opened along the greater curvature, washed in saline, and compressed between glass slides for better viewing. The slides were scanned at 1200 dpi. The percentage area of gastric lesions (glandular portion) was determined with the aid of ImageJ software. The injured area is expressed as a relative percentage of the total area of the gastric body [23, 26].

### 2.9. Effect of HELCC on Intestinal Motility [27]

The animals were treated with HELCC (75 mg/kg, p.o.), vehicle (saline, 0.1 mL/10 g, p.o.), or atropine (0.01 g/kg, p.o.) and 10% activated charcoal (0.1 mL/10 g, p.o.). HELCC, atropine, or vehicle was administered first, followed by activated charcoal 1 h later. Thirty minutes after charcoal administration, the animals were sacrificed, and their small intestine was removed. The total length of the intestine (the pyloric region to the ileocecal junction) was then measured; the distance traveled by the charcoal was determined based on the distance from the pylorus to the last portion of the intestine that contained at least 1 cm of continuous charcoal. Thirty minutes after administration of the charcoal, the animals were sacrificed and analyzed as described above.

### 2.10. Statistical Analysis

Data are presented as the mean ± standard deviation. The level of significance was 0.05. Differences between the means were determined by analysis of variance (ANOVA), and the Newman-Keuls test was used to determine statistical significance. Analyses were performed using GraphPad Prism software version 5.0.

## 3. Results

Phytochemical prospecting [20] of HELCC identified the presence of tannins flavonols, flavones, flobabenics, flavanone, flavonals, xanthones, terpenes, and alkaloids (Table 1), possibly involved in the bioactive properties evaluated.

### 3.1. Toxicological Data

HELCC did not show evidence of acute toxicity. The daily monitoring of weight of animals did not indicate a significant variation compared to the control group (saline). There were no signs of morbidity or death just after extract administration, or during the period of observation and weighing (14 days after treatment) in the group treated orally. The median lethal dose (LD50) for the oral administration of HELCC was greater than or equal to 5000 mg/kg, with a 95% confidence interval.

 The animals showed decreased activity and mild dyspnea, showing recovery within four hours.

### 3.2. Gastroprotection Tests

**Figure 1:**
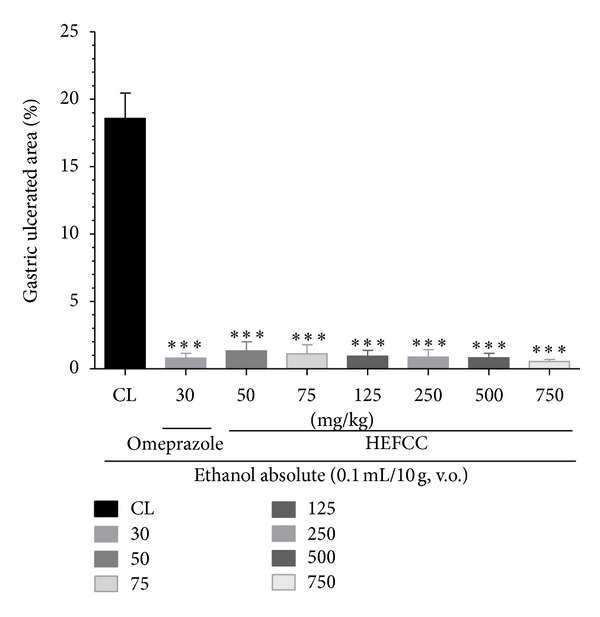
Effect of oral administration of the HEFCC in gastric lesions induced by ethanol abs in mice. Significance***P < 0.001 when compared with control group.

The effects of HELCC on gastric lesions induced by absolute ethanol (0.2 mL/animal, p.o.) are shown in Figure 1. The animals that received only vehicle combined with oral administration of absolute ethanol showed an extensive area of gastric lesion (18.57 ± 1.87%). HELCC produced a marked reduction in the area damaged by absolute ethanol at all doses tested: 1.34 ± 0.66% at 50 mg/kg (92.78% reduction), 1.11 ± 0.67% at 75 mg/kg (94.02%), 0.93 ± 0.43% at 125 mg/kg (94.99%), 0.87 ± 0.54% at 250 mg/kg (95.31%), 0.82 ± 0.31% at 500 mg/kg (95.58%), and 0.53 ± 0.16% at 750 mg/kg (97.14%), when compared to the control (*P* < 0.001 in all cases). The animals that received omeprazole (30 mg/kg, p.o.) also showed a significant decrease in gastric lesion area and a substantial reduction percentage in ulcer area, that is, 0.78 ± 0.34% and 95.79%, respectively, compared to control (*P* < 0.001).

**Figure 2:**
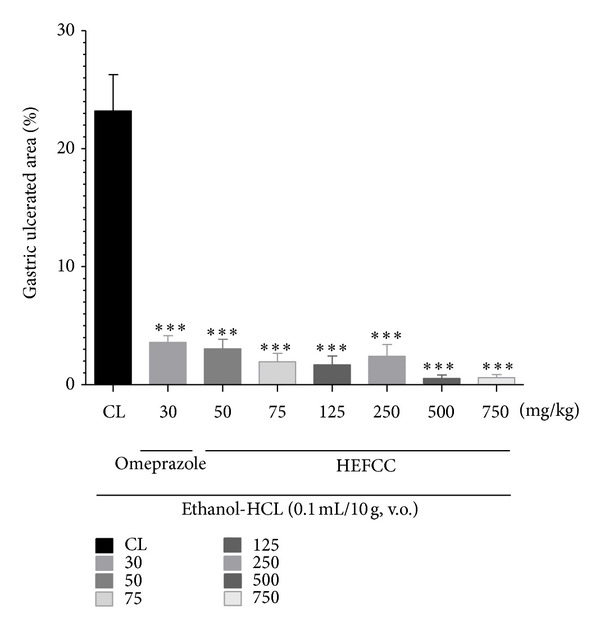
Effect of oral administration of the HEFCC in gastric lesions induced by acid ethanol in mice. Significance ***P < 0.001 when compared with control group.

The effects of HELCC on gastric lesions induced by acidified ethanol (0.2 mL/animal, p.o.) are shown in Figure 2. The animals that received only vehicle combined with oral administration of acidified ethanol showed an extensive area of gastric lesion (23.19 ± 3.09%). HELCC showed substantial reduction in the areas damaged by acidified ethanol, at all doses tested: 3.04 ± 0.80% at 50 mg/kg (reduction of 86.89%), 1.94 ± 0.70% at 75 mg/kg (91.63%), 1.67 ± 0.75% at 125 mg/kg (92.79%), 2.40 ± 0.99% at 250 mg/kg (89.65%), 0.52 ± 0.30% at 500 mg/kg (97.75%), and 0.59 ± 0.26% at 750 mg/kg (97.45%), respectively, when compared to the control (*P* < 0.001 in all cases). The animals that received omeprazole (30 mg/kg, p.o.) also showed a significant reduction in the injured areas and a high percentage reduction in ulcer area, that is, 3.58 ± 0.56% and 84.56%, respectively, compared to control (*P* < 0.001).

**Figure 3:**
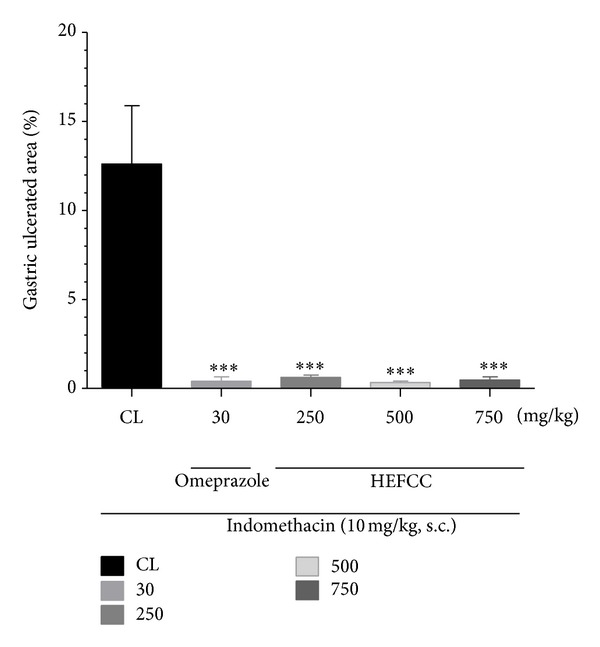
Effect of oral administration of the HEFCC in gastric lesions induced by indomethacin in mice. Significance***P < 0.001 when compared with control group.

 The effects of HELCC on gastric lesions induced by indomethacin (10 mg/kg, s.c.) are shown in Figure 3. The animals that received only vehicle combined with s.c. administration of indomethacin showed a large area of gastric lesion (12.61 ± 3.27%). HELCC produced a significant reduction in the areas damaged by indomethacin, at all doses tested: 0.63 ± 0.11% at 250 mg/kg (reduction of 95.00%), 0.33 ± 0.08% at 500 mg/kg (97.38%), and 0.47 ± 0.17% at 750 mg/kg (97.38%), respectively, when compared to the control (*P* < 0.001 in all cases). The animals that received omeprazole (30 mg/kg p.o.) also showed significant reduction in gastric lesion area and a substantial percentage reduction in ulcer area, that is, 0.40 ± 0.24% and 96.82%, respectively, compared to control (*P* < 0.001).

**Figure 4:**
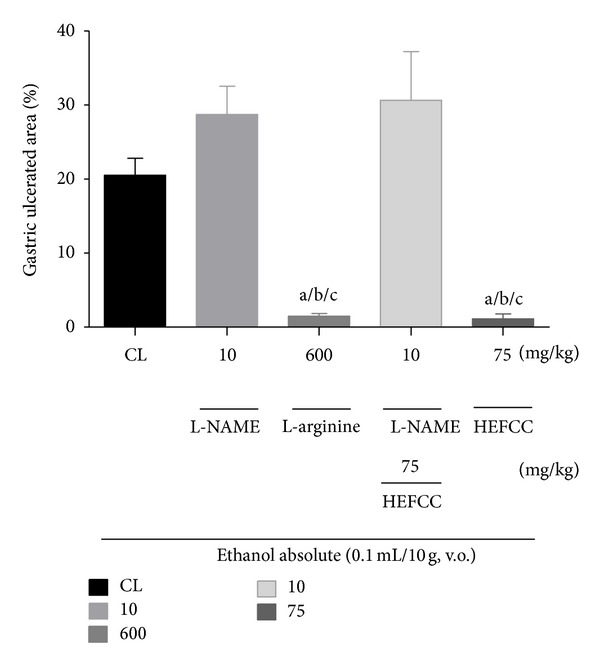
The role of nitric oxide (NO) in the gastroprotector effect of the HEFCC in gastric lesions model induced by ethanol absolute in mice. Significance ^a^ *P* < 0.001 versus CL, ^b^ *P* < 0.001 versus L-NAME (10 mg/Kg, v.o.), and ^c^ *P* < 0.001 versus L-NAME (10 mg/kg, v.o.) + EHFCC (75 mg/kg, v.o.).

In the evaluation of the role of nitric oxide (NO) in the protective effect of HELCC against gastric lesions induced by absolute ethanol in mice, it was observed that the animals that received only vehicle combined with oral administration of absolute ethanol showed gastric lesion (20.52 ± 2.29%). The animals that received L-NAME (10 mg/kg, i.p.), an inhibitor of nitric oxide synthase (NOS), along with absolute ethanol (0.2 mL/animal, p.o.), also showed a large percentage of ulcerated area (28.73 ± 3.83%). However, the animals that received L-arginine (600 mg/kg, p.o.) in combination with absolute ethanol exhibited reductions in injured area (1.47 ± 0.38%), with percentage of ulcer reduction of 92.83%. HELCC (75 mg/kg, p.o.) produced a significant (*P* < 0.001) reduction in ulcerated area to 1.11 ± 0.67% or a percentage reduction of 94.59%, compared to control. However, HELCC (75 mg/kg, p.o.), when given with L-NAME (10 mg/kg, i.p.), had its effect blocked, resulting in a percentage of ulcerated area of 30.65 ± 6.55% (Figure 4).

**Figure 5:**
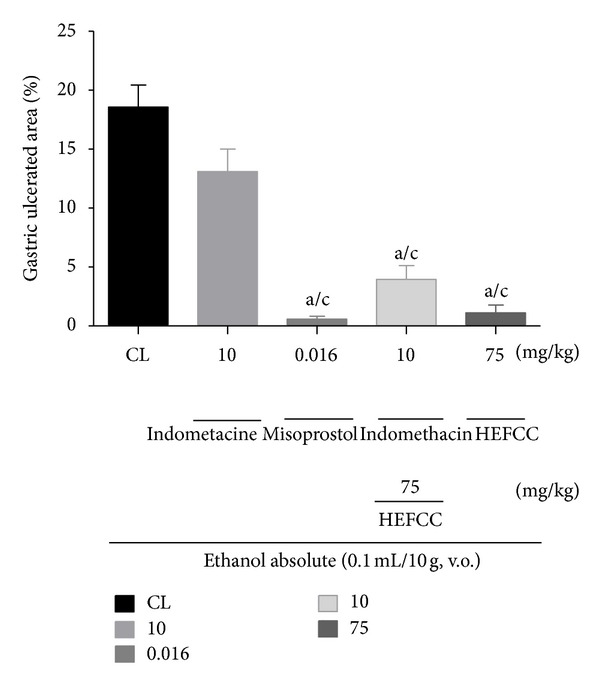
The role of prostaglandins in gastroprotector effect of the HEFCC in gastric lesion model induced by ethanol absolute in mice. Significance ^a^ *P* < 0.001 versus CL, ^b^ *P* < 0.01 versus CL, ^c^ *P* < 0.001 versus indomethacin (10 mg/Kg, s.c.).

In the investigation of the role of prostaglandins in the protective effect of HELCC against gastric lesions induced by absolute ethanol in mice, it was seen that the animals that received only vehicle in combination with oral administration of absolute ethanol showed a gastric lesion area of 18.57 ± 1.87%. The animals that received indomethacin (10 mg/kg, s.c.) together with absolute ethanol (0.2 mL/animal, p.o.) displayed a percentage of ulcerated area of 13.09 ± 1.92%. However, the animals that received misoprostol (0.016 mg/kg, p.o.) combined with absolute ethanol showed reductions in injured area (0.57 ± 0.25%), resulting in a percentage reduction of 96.95%. HELCC (75 mg/kg, p.o.) produced a significant (*P* < 0.001) reduction in ulcerated area to 1.11 ± 0.67% or a percentage reduction of 94.02%, compared to the control. HELCC (75 mg/kg, p.o.) when combined with indomethacin (10 mg/kg, s.c.) resulted in a percentage of ulcerated area of 3.95 ± 1.15% and a percentage reduction in ulcer area of 78.72% (Figure 5).

**Figure 6:**
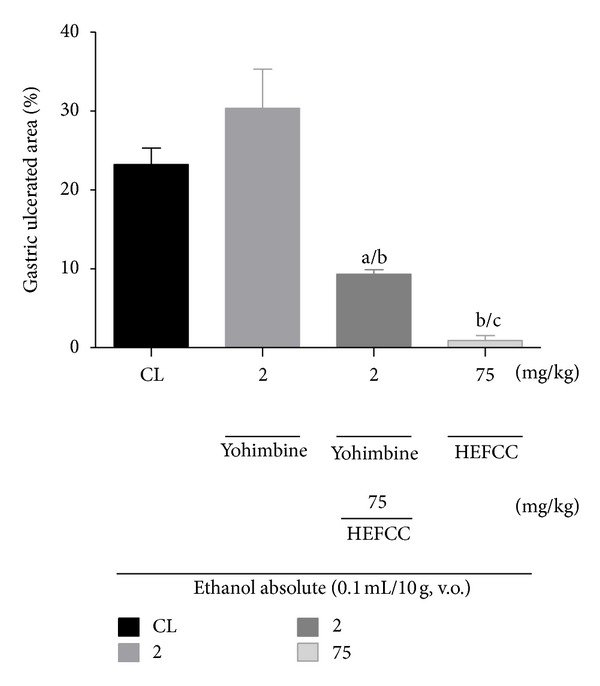
The role of noradrenergic receptor alpha_2_ in the gastroprotector effect of HEFCC in gastric lesion model induced by ethanol abs in mice. Significance ^a^ *P* < 0.001 versus CL, ^b^ *P* < 0.001 versus glibenclamide (5 mg/kg, i.p.).

In the investigation of the role of the alpha-2 noradrenergic receptor in the protective effect of HELCC against gastric lesions induced by absolute ethanol in mice, it was seen that the animals that received only vehicle combined with oral administration of absolute ethanol showed a gastric lesion area of 23.22 ± 2.08%. The animals that received yohimbine (2 mg/kg, i.p.) together with absolute ethanol (0.2 mL/animal, p.o.) exhibited an extensive ulcerated area of 30.34 ± 4.96%. HELCC (75 mg/kg, p.o.) produced a significant (*P* < 0.001) reduction in ulcerated area to 0.91 ± 0.63% or a percentage reduction of 96.34%, compared to the control. Also, HELCC (75 mg/kg, p.o.) given together with yohimbine (2 mg/kg, i.p.) produced a significant (*P* < 0.01) gastroprotective effect with a percentage of ulcerated area of 9.30 ± 0.59% or a percentage reduction in ulcer area of 59.94% (Figure 6).

**Figure 7:**
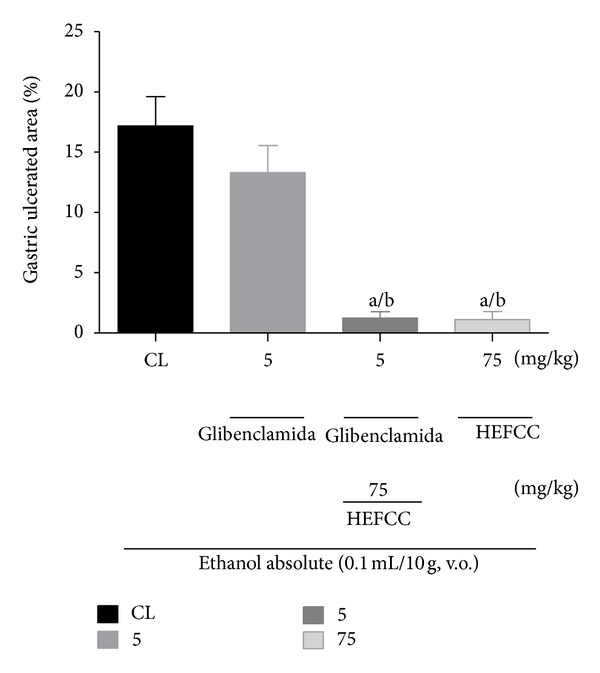
The role of ATP-dependent K+ channels in the gastroprotector effect of HEFCC in gastric lesion model induced by ethanol absolute in mice. Significance ^a^ *P* and ^a^ *P* < 0.001 versus CL, ^b^ *P* < 0.001 versus glibenclamide (5 mg/kg, i.p.).

In the evaluation of the involvement of ATP-dependent K^+^ channels in the protective effect of HELCC against gastric lesions induced by absolute ethanol in mice, it was observed that the animals that received only vehicle in combination with oral administration of absolute ethanol displayed a gastric lesion area of (17.17 ± 2.44%). The animals that received glibenclamidee (5 mg/kg, i.p.) along with absolute ethanol (0.2 mL/animal, p.o.) showed an ulcerated area of 13.30 ± 2.25%). HELCC (75 mg/kg, p.o.) produced a significant (*P* < 0.001) reduction in ulcerated area to 1.11 ± 0.67% or percentage reduction of 91.65%, compared to the control. Also, HELCC (75 mg/kg, p.o.) when given with glibenclamidee (5 mg/kg, i.p.) still exerted a significant gastroprotective effect (*P* < 0.001) with a percentage of ulcerated area of 1.23 ± 0.53% or percentage reduction in ulcer area of 90.75% (Figure 7).

### 3.3. Evaluation of Intestinal Motility

**Figure 8:**
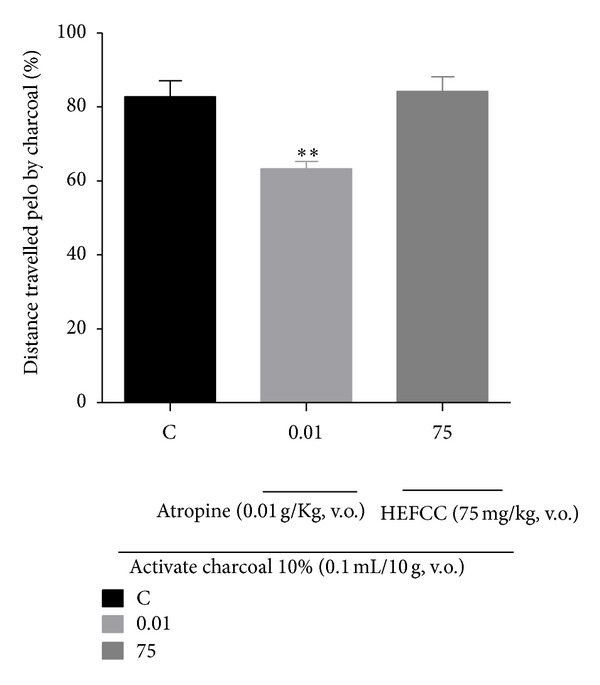
The effect of HEFCC on intestinal motility in mice. Significance **P < 0.01 versus C (vehicle control).

The percentage of the distance traveled by the marker (activated charcoal) in the small intestine of mice was (82.77 ± 4.29%). The animals that received HELCC (75 mg/kg) orally had a distance traveled by the marker (84.20 ± 3.94%) that was similar to that obtained by vehicle group, demonstrating that HELCC did not significantly affect gastrointestinal motility. However, in the group treated with atropine (0.01 g/kg, p.o.), a muscarinic antagonist, there was a decrease in intestinal motility with a distance traveled of 63.21 ± 2.05%, compared to the vehicle control group (saline, p.o.), showing a significant effect (*P* < 0.01), as seen in the data presented in Figure 8.

## 4. Discussion

Several species of the genus *Croton *have played an important role in traditional uses of medicinal plants in Africa, Asia, and South America. Such uses include the treatment of cancer, constipation, diarrhea and other digestive problems, external wounds, diabetes, dyslipidemia, hypertension, fever, inflammation, intestinal worms, malaria, pain, ulcers, and obesity [9]. In the toxicological evaluation, HELCC did not show signs of significant acute toxicity in the animals tested and at all doses administered, corroborating a previous study that demonstrated the low acute toxicity of *Croton campestris* A. St.-Hill. in two guinea pigs [28].

As seen in the evaluation of the gastroprotective activity of HELCC, experimental animals play an important role in the search for new drugs with protective properties. Considering that the etiology of ulcer is multifactorial, lesions in the gastric mucosa can be induced in different experimental models through various mechanisms [29]. Some of the most used acute models for the evaluation of antiulcerogenic substances in animals are the models of gastric lesion induced by absolute ethanol or indomethacin [30]. In this work, besides the use of these methodologies, the effects of HELCC were also evaluated with the gastric ulceration model using acidified ethanol.

Ethanol-induced gastric ulcer occurs predominantly in the glandular portion of the stomach, as a result of a direct necrotizing action and, moreover, the lack of defense factors such as secretion of bicarbonate and mucus and increased oxidative stress [31]. HELCC at all doses tested by oral administration prevented, in a dose-dependent manner, gastric lesion induced by the administration of absolute ethanol. The doses decreased the percentage of gastric ulcer area in the same manner as with omeprazole, a well-known proton pump inhibitor, when compared to the vehicle-treated control lesion group. It was also demonstrated that the gastroprotective action of HELCC at all orally administered doses, against gastric ulcer induced by acidified ethanol, resulted in a percentage reduction in ulcer area similar to that with omeprazole, in comparison with the vehicle-treated control lesion group. 

Indomethacin like the majority of nonsteroidal anti-inflammatory* drugs* (NSAIDs) acts as an inhibitor of prostaglandin synthesis and consequently diminishes the defense mechanisms of gastric mucus [32–34]. It is the primary mechanism by which this class of drugs cause, damage to the gastrointestinal tract [35].

This research demonstrates the ability of the HELCC to inhibit the gastric damage caused by NSAIDs, where we used a classic model of induction of gastric lesion by s.c. indomethacin. Orally administered HELCC, at the doses tested, was able to prevent the appearance of gastric lesions in animals subjected to the treatment with this NSAID, showing significant results when compared to the gastric lesion control.

 Among the various factors involved in maintaining the integrity of the gastric mucus and protection against injury caused by ulcerogenic agents, we examined the contribution of endogenous nitric oxide, prostaglandins, ATP-sensitive potassium channels (KATP), and alpha-2 noradrenergic receptor [36, 37] to the action of HELCC on the gastric mucosa.

The discovery of nitric oxide (NO) as an agent of cell signaling was one of the most important events in human physiology of the last 80–90 years. NO is a free radical gas, which has drawn attention because of its role in signal transduction related to various physiological processes such as smooth muscle relaxation and vasodilation, neurotransmission, platelet aggregation mechanisms, regulation of pro- and antiapoptotic mechanisms, and control of blood pressure and blood flow [38, 39].

Related to the modulation of the physiological components of gastrointestinal tract, NO plays an important role in the control of gastrointestinal motility [40], gastric blood flow [41], recruitment of neutrophils [42], and secretion of mucus [43]. 

In the present research, the assessment of the role of NO in the gastroprotective effect of HELCC in models of gastric lesions, induced by ethanol in mice, showed that the animals that received L-NAME (a nonspecific inhibitor of NO synthase), showed a large ulcerated gastric area, after administration of absolute ethanol, as in the control lesion group. HELCC managed to inhibit the appearance of gastric lesions in the gastroprotection model using L-NAME. The extract in this situation showed a non-significant percentage reduction in ulcer area. Similarly, it was seen that the L-arginine (a substrate of NO synthase) significantly reduced the percentage of ulcerated gastric area when compared with the vehicle-treated control lesion group. This suggests that the compounds present in the extract that exert a gastroprotective effect involving the cytoprotective effect of NO. 

Considering the traditional use of the leaves of the species *Croton campestris* A. St.-Hill. for gastric disturbances, in the form of teas and infusion, and in view of the presence of such secondary metabolites as tannins, flavonoids, and alkaloids in the extract, it is possible to correlate the antiulcerogenic effect demonstrated by the plant with these compounds. Also, it is possible to correlate studies that demonstrate an association between plant species that contain tannins, flavonoids, and alkaloids and gastroprotective effects [44, 45], as probably occur with *Croton campestris* A. St.-Hill.

The protective action of prostaglandins (PGs) on the gastric mucosa is mediated by an increase in mucus production and secretion of bicarbonate, modulation of gastric acid secretion, inhibition of the release of inflammatory mediators by mast cells, and the maintenance of blood flow during exposure to irritants. It is known that prostaglandin E2 (PGE2) has a gastroprotective action against lesions caused by ethanol and that this protection stems from an increase in intracellular guanosine-3′, 5′-cyclic monophosphate (cGMP), which is mediated by an increase in intracellular free calcium concentration and nitric oxide production [46].

In the evaluation of a possible involvement of prostaglandins in the gastroprotective effect of HELCC, we used misoprostol, an analogue of prostaglandin E1 (PGE1), as well as indomethacin, and an inhibitor of PG synthesis. Misoprostol significantly inhibited the development of gastric lesions when compared with the vehicle-treated control lesion group. Pretreatment with indomethacin did not reverse the cytoprotective effect of HELCC, demonstrating just a trend in this direction. On the basis of this result, we cannot state that prostaglandins play a role in the relaxant effect of HELCC.

The modulation of *α*2 receptors located in the intramural peripheral parasympathetic ganglia decreases the discharge of vagal acetylcholine, which reduces gastric secretion and motility and increases blood flow. The *α*2-noradrenergic receptors and the *α*2 presynaptic are involved in the regulation of gastric acid secretion and are effective in protection against chemical agents such as NSAIDs and ethanol, where their effects may be mediated central and peripheral receptors [47].

In our study, the administration of yohimbine (10 mg/kg, i.p.), an indole alkaloid that promotes the release of neurotransmitters by blocking the presynaptic *α*2 receptors [48], did not reverse the HELCC effect. This indicates that the gastroprotective effect of HELCC does not act by modulation of *α*2 receptor activity.

KATP channels are regulated by ligands and their mechanism is defined based on their sensitivity to intracellular ATP, which inhibits its activity. It has been postulated that KATP channels are involved in a variety of pathophysiological functions in the stomach such as regulation of blood flow, gastric acid secretion, and gastric muscle contractility [49]. In the vascular system, these channels are related to the relaxation of vascular smooth muscle, having an important role in blood pressure control. This vasodilation can be blocked by glyburide, a sulfonylurea that blocks KATP channels [50]. Our studies showed that HELCC, when combined with glibenclamidee, significantly retained its gastroprotective effect, indicating that the mechanism of action of the active ingredients of the extract may not involve the stimulation of KATP channels.

Another way in which HELCC could promote protection of the gastric mucosa would be through increased gastrointestinal motility, so increased intestinal transit would accelerate gastric emptying, thereby decreasing the effect of aggressor acid in the stomach and duodenum. Cholinergic innervation of the circular muscle layer of TGI, acting on muscarinic receptors M1 and M3, is primarily responsible for gastrointestinal motility.

 In this sense, HELCC did not significantly alter the intestinal transit of mice when compared to the vehicle group (10% activated charcoal) and the group that received atropine, which is a drug blocking the muscarinic action of acetylcholine. 

## 5. Conclusion

In short, we demonstrate here for the first time that HELCC, an extract of leaves of *Croton campestris A. St.-Hill.,* has a protective effect against acute gastric lesion induced by absolute ethanol, acidified ethanol, or indomethacin in mice, thus indicating a cytoprotective action of HELCC, which occurs through the potentiation of the NO/cGMP pathway. The leaves of *C. campestris *contain compounds (metabolites) that possibly act in synergy in the activation of defense factors and in reducing aggressor factors of gastric mucosa, which makes this extract promising for the development of new therapies to combat gastropathy associated with NSAID-induced peptic ulcer disease.

## Figures and Tables

**Table 1:** Prospecting photochemistry of hydroalcoholic extract of lyophilized of leaves of *Croton campestris *A. St.-Hill.

Metabolites	(+) presence (−) absence
Phenols	**−**
Pyrogallic tannins	**−**
Flobabenic* tannins *	**+**
Anthocyanins	**−**
Anthocyanidins	**−**
*Flavones *	**+**
*Flavonols *	**+**
*Xanthones *	**+**
Chalcones	**−**
Aurones	**−**
*Flavanonols *	**+**
Leucoanthocyanidin	**−**
Catechin	**−**
*Flavonone *	**+**
*Alkaloids *	**+**
*Terpenes *	**+**
Steroids	**−**
